# Effectiveness of the mHealth intervention ‘MyDayPlan’ to increase physical activity: an aggregated single case approach

**DOI:** 10.1186/s12966-021-01163-2

**Published:** 2021-07-07

**Authors:** L. Degroote, A. De Paepe, I. De Bourdeaudhuij, D. Van Dyck, G. Crombez

**Affiliations:** 1 Department of Movement and Sport Sciences, Ghent University, Ghent, Belgium; 2 Research Foundation Flanders, Brussels, Belgium; 3 Department of Clinical-Experimental and Health Psychology, Ghent University, Ghent, Belgium

## Abstract

**Background:**

e- and mHealth interventions using self-regulation techniques like action and coping planning have the potential to tackle the worldwide problem of physical inactivity. However, they often use one-week self-regulation cycles, providing support toward an active lifestyle on a weekly basis. This may be too long to anticipate on certain contextual factors that may fluctuate from day to day and may influence physical activity. Consequently, the formulated action and coping plans often lack specificity and instrumentality, which may decrease effectiveness of the intervention. The aim of this study was to evaluate effectiveness of a self-regulation, app-based intervention called ‘MyDayPlan’. “MyDayPlan’ provides an innovative daily cycle in which users are guided towards more physical activity via self-regulation techniques such as goal setting, action planning, coping planning and self-monitoring of behaviour.

**Methods:**

An ABAB single-case design was conducted in 35 inactive adults between 18 and 58 years (M = 40 years). The A phases (A1 and A2) were the control phases in which the ‘MyDayPlan’ intervention was not provided. The B phases (B1 and B2) were the intervention phases in which ‘MyDayPlan’ was used on a daily basis. The length of the four phases varied within and between the participants. Each phase lasted a minimum of 5 days and the total study lasted 32 days for each participant. Participants wore a Fitbit activity tracker during waking hours to assess number of daily steps as an outcome. Single cases were aggregated and data were analysed using multilevel models to test intervention effects and possible carry-over effects.

**Results:**

Results showed an average intervention effect with a significant increase in number of daily steps from the control to intervention phases for each AB combination. From A1 to B1, an increase of 1424 steps (95% CI [775.42, 2072.32], t (1082) = 4.31,*p* < .001), and from A2 to B2, an increase of 1181 steps (95% CI [392.98, 1968.16], t (1082) = 2.94, *p* = .003) were found. Furthermore, the number of daily steps decreased significantly (1134 steps) when going from the first intervention phase (B1) to the second control phase (A2) (95% CI [− 1755.60, − 512.38], t (1082) = − 3.58, *p* < .001). We found no evidence for a difference in trend between the two control (95% CI [− 114.59, 197.99], t (1078) = .52, *p* = .60) and intervention phases (95% CI [− 128.79,284.22], t (1078) = .74, *p* = .46). This reveals, in contrast to what was hypothesized, no evidence for a carry-over effect after removing the ‘MyDayPlan’ app after the first intervention phase (B1).

**Conclusion:**

This study adds evidence that the self-regulation mHealth intervention, ‘MyDayPlan’ has the capacity to positively influence physical activity levels in an inactive adult population. Furthermore, this study provides evidence for the potential of interventions adopting a daily self-regulation cycle in general.

**Supplementary Information:**

The online version contains supplementary material available at 10.1186/s12966-021-01163-2.

## Background

The Global Burden of Disease Study (GBD) estimated that worldwide over 72% of deaths and almost 61% of disability-adjusted life years (DALYs), the number of years lost due to ill-health, disability or early death, are attributed to non-communicable diseases (NCDs) such as cardiovascular diseases (heart attacks and stroke), cancers, chronic respiratory diseases and diabetes [1]. Physical inactivity is one of the major risk factors for NCDs that, to a large extent, can be avoided [2]. In 2018, it was stated in The Lancet Global Health that more than one in four adults worldwide (28% or 1.4 billion people) are physically inactive [3], meaning that they do not meet the health recommendations of at least 150 min of moderate-intensity physical activity, or at least 75 min of vigorous-intensity physical activity throughout the week, or an equivalent combination of both [3]. Although this physical activity recommendation is currently the norm, physical inactivity could also be defined as not meeting the equivalent step recommendation to accumulate 10000steps/day [4]. However, this 10000steps guideline is not a universal guideline for all populations. These inactivity rates indicate the need for lifestyle interventions that effectively promote physical activity in a large number of people at low [5, 6].

Mobile health (mHealth) technology has potential to serve as a platform for physical activity promotion interventions. mHealth is part of eHealth, and refers to the use of mobile communication devices, such as mobile phones, tablet computers and personal digital assistants (PDAs), and wearable devices (e.g. smart watches) in support of health and health-related fields (i.e. healthcare, health surveillance and health education, knowledge and research) [7]. The potential value of mHealth lies in its widespread appeal, accessibility and ability to reach large populations at a low cost [8]. It also offers the possibility of tailoring interventions to the specific needs of individuals or groups. Therefore, the use of mHealth has been widely adopted with for example more than 100,000 health-related apps on the mobile phone app market [9]. According to the World Health Organization, 112 countries have reported the existence of at least one mHealth initiative [10]. Several systematic reviews and meta-analysis evaluated the effectiveness of existing mHealth interventions on the level of physical activity revealing a potential to increase physical activity in the short term [11–19]. Of course, the effectiveness of these interventions can not only be attributed to the use of e- and mHealth strategies, but mainly depends on the content of these interventions. In that context, previous research has shown that theory-based interventions are more effective to change behaviour than atheoretical interventions [20–22]. For example, interventions based on the Health Action Process Approach (HAPA) have already been effective in altering health behaviours such as physical activity and dietary behaviour [23–29]. Interventions grounded within the Health Action Process Approach adopt self-regulation techniques such as goal setting, action planning, coping planning and self-monitoring [30]. Pre-intentional processes are often addressed by providing personal feedback to raise awareness and motivate participants to change their behaviour. Post-intentional processes that aim to overcome the gap between intentions and behaviour are often addressed by inviting participants to make an action plan, by offering the possibility to identify difficult situations, hindering factors and the relevant solutions to overcome these (i.e. coping planning). Finally, self-monitoring behaviour is often used to contribute in pursuing their health goals [31].

So, HAPA based mHealth interventions seem to have the potential to effectively increase the level of physical activity. However, an important challenge remains: earlier HAPA based interventions often used self-regulation cycles lasting for a few days, a week or even a month meaning that goals, action plans and coping plans were set for these coming days, this week, this month [26, 29, 32–35]. Although these timescales are certainly relevant, they may be too long to incorporate the dynamic context of the upcoming week and to formulate highly specific and instrumental action and coping plans. This was confirmed by earlier qualitative research revealing that users found it difficult to formulate goals, actions and coping plans for the coming week as it was not possible to predict and anticipate on certain contextual factors that may fluctuate from day to day (e.g. location, weather, agenda, mood, health status, motivation, …) [36, 37]. Consequently, the set weekly action and coping plans lacked specificity (specific plans are plans that are clearly defined and leave no room for interpretation) and instrumentality (instrumental plans can serve as a crucial and effective mean or tool) [38].

This challenge was addressed during the development of the ‘MyDayPlan’ intervention. ‘MyDayPlan’ is a HAPA based mHealth intervention aiming at increasing the level of physical activity in a general adult population. ‘MyDayPlan’ implemented a daily self-regulation cycle, allowing users to set more achievable, realistic goals, action and coping plans by taking into account the anticipated opportunities/obstacles of the day. Furthermore, short (daily) cycles of action and coping planning allow individuals to ‘learn by doing’, meaning that they may gradually optimize the quality (specificity and instrumentality) of their action and coping plans based on own experience.

The aim of this study is to evaluate the effectiveness of ‘MyDayPlan’ on the level of physical activity, more specifically the amount of daily steps, in a general adult population. Following hypotheses are formulated: (1) we hypothesize that the use of ‘MyDayPlan’ will increase the number of daily steps and that the number of daily steps will decrease when participants stop using the app. (2) Although we expect a decrease in the number of steps when participants stop using the app, we hypothesize that a carry-over effect will be seen. This means that after a period of using the app, the number of daily steps would still be higher during the first few days after removing the ‘MyDayPlan’ app compared to baseline level. This hypothesis is based on the idea that, after ‘mandatory’ exposure to the self-regulation techniques of ‘MyDayPlan’, users potentially would adopt techniques such as action and coping planning even without using the app.

## Methods

An ABAB single-case design in combination with multilevel-modelling analysis were used to address these hypotheses.

### Participants

An eligible sample of men and women between 18 and 65 years old was recruited using purposive sampling. Participants were individually addressed and recruited in the city library of Ghent, which is also a meeting place, cultural centre and research site, and via Facebook, flyers, posters and word-of-mouth (snowball sampling). Inclusion criteria were age, having no current medical and physical limitations, owning a smartphone using Android as operating system, having Internet access, having the ability to understand and speak Dutch. The main aim of the ‘MyDayPlan’ intervention is to guide physically inactive adults towards engaging in sufficient levels of PA in order to take advantage of the positive health consequences related with it. Therefore an exclusion criterium to be included in this study was achieving the physical activity guidelines of 150 min of moderate-to-vigorous physical activity per week. Furthermore, to avoid influences of earlier experiences with monitoring PA, already monitoring steps or level of physical activity using a smartphone or other wearable device was also included as an exclusion criterium. When people showed interest in the study, they were asked to fill out a questionnaire (via email) assessing their demographic characteristics and whether they met the inclusion and exclusion criteria. To assess their current level of physical activity, the International Physical Activity Questionnaire (IPAQ, long 7d version) was used as a screening tool [39].

Based on the questionnaire results, only people who met the inclusion criteria were included in the study. In total 127 adults filled out the questionnaire, resulting in a sample of 35 eligible participants that were included in the study. Of the 92 participants that were excluded, 58 were excluded because they already achieved the physical activity guidelines of 150 min of moderate-to-vigorous physical activity per week (IPAQ uses 30 min of MVPA per day). MVPA levels within these 58 participants varied from 30.52 min of MVPA per day to 210.69 min of MVPA per day. Furthermore, 13 participants were excluded because they already monitored steps or level of physical activity using a smartphone or other wearable device, and 21 were excluded because of both reasons. All participants read and signed an informed consent form. This study was approved by the Ethics Committee of the University hospital of Ghent (study number: B670201938577).

### Description of MyDayPlan

‘MyDayPlan’ is an mHealth intervention targeting physical activity, using a mobile application in combination with a Fitbit activity monitor. A daily self-regulation cycle was implemented in ‘MyDayPlan’ to guide users towards more physical activity. Furthermore, as implementing these daily assessment moments (1 day self-regulation cycles) could increase participant burden, several measures were taken to avoid attrition. The assessment moments were kept as short as possible, only including some key self-regulation techniques. Also, during development, time-efficiently and ease of use of the app were taken into account. Before evaluating its effectiveness, a qualitative study in which semi-structured interviews were conducted with potential end-users evaluated acceptability and feasibility of ‘MyDayPlan’ [40]. This study revealed that ‘MyDayPlan’ is well-received and seems to be feasible and acceptable in a general adult population. However, based on the recommendations coming from the users, several adaptations were made to ‘MyDayPlan’: an activity tracker was implemented to self-monitor physical activity, intuitiveness was increased by minimizing text input and providing more pre-programmed options and finally, more guidance was provided for the coping planning component by providing more tailored examples. This resulted in the current, second version of the ‘MyDayPlan’ app .

In ‘MyDayPlan’, participants go through the same cycle each day, offering self-regulation techniques as follows: each morning at 8 am, participants receive a notification to go to the app. When responding, they are automatically directed towards the “goal” module, in which they are requested to set a specific step goal, taking into account the anticipated possibilities or obstacles of that day. Within ‘MyDayPlan’ it was chosen to use ‘step goals’ instead of ‘active time (MVPA) goals’ because of two reasons: first, by encouraging users to set step goals and specific step related actions, performing the actions will directly reflect in the number of daily steps. Which is not necessarily the case with MVPA for example, as it is very difficult for users to estimate which activities are MVPA and which are not. Second, earlier research has revealed that the current physical activity trackers accurately assess number of daily steps, but do not accurately assess time of MVPA [41]. As ‘MyDayPlan’ included the implementation of an activity tracker, it made sense to choose number of daily steps as the measure of physical activity. To give participants some guidance during the first time they set a step goal, some optional information and guidelines on steps are provided in the app.

After setting a step goal, they are required to select or formulate specific actions that they plan to do that day to achieve their set step goal. They have the possibility to select or formulate specific actions within 4 domains (transport, household, work/school, leisure time). For each domain they can choose to select predetermined actions (e.g. mow the lawn, use the coffee machine furthest from my work spot, …) or they can choose to formulate own actions.

As a next step, after setting/formulating actions, users are required to formulate possible barriers and possible solutions to overcome these (coping planning). They again have the choice to select predetermined barriers and solutions or to formulate other ones. After this, the morning session is finished.

For the evening session, users receive a notification at 8 pm after which they have access to initiate the evening session. They are again required to go to the app and to look back on their set goal. More specifically they are required to fill in the amount of steps they have taken that day, based on the measurement of the Fitbit they are wearing. Automatically the app calculates whether they have reached their set step goal or not. When they have met their goal, they are invited to think about the factors that have helped to reach their goal and to reflect on these as possible solutions for barriers the next day. If they did not meet their goal, users are required to think about the factors that have prevented them from achieving their goal. They are required to reflect on these as barriers for the next day. After this, the evening session is finished. Table 1 gives an overview of the behaviour change techniques that are part of the app. Screenshots of ‘MyDayPlan’ are added as a supplementary file (supplementary file 1).

**Table 1:** Overview of the self-regulation techniques implemented in the MyDayPlan app

Self-regulation Technique	Implementation mode
Goal setting	Participants set a step goal each morning.
Action planning	Participants select or formulate specific actions to achieve their step goal.
Barrier identification/problem solving (=coping planning)	Participants select and/or formulate specific barriers and possible solutions to overcome these barriers.
Self-monitoring of behaviour	Participants can monitor their steps using the Fitbit activity tracker.
Prompting review of behavioural goals	Participants reflect on whether they achieved their step goal or not. They also reflect on what helped to achieve the goal or on what hindered them not to reach their goal.

### Study design and procedure

In order to adequately test the set hypotheses, an ABAB design, also called withdrawal or reversal design was performed. This single-case design has four phases denoted by A1, B1, A2, and B2. In each phase, repeated measurements of the participant’s behaviour are obtained. Hence, the participant serves as his or her own control. The A phases are the control phases, the B phases are the intervention phases. Both phases are presented twice to the participants. A1, is used to establish a baseline for the behaviour. After this baseline phase, the intervention is presented for the first time during B1. In the third phase, A2, the intervention is withdrawn. After this phase, the intervention is presented a second time, during the B2 phase [42]. This within-subject design was chosen over a more standard between-subject randomized-controlled trial (RCT) design because of several reasons [43]. First, the design allows making a large number of repeated observations, enabling a detailed study of the evolution of the behaviour within individuals. Second, given the large number of repeated measurements within subject, less participants are needed to achieve sufficient statistical power compared to between-subject designs, making this design more cost efficient. Third, every participant takes part in the active components of the intervention in contrast to RCTs where half of the participants is assigned to a control group. A possible drawback that is often mentioned is the risk of carryover effects from the intervention period to the subsequent periods. However, in the present study we were particularly interested in these carryover effects as it could reflect long-term effects of using the app.

The study duration was 32 days for all participants. The duration of the 4 different phases of the design however, varied between participants, but each phase had a duration of minimum 5 days. The randomization of phase length was done to avoid influence of the start day of the different phases and of the weekly PA pattern that is typically shown (e.g. the so called “weekend warriors” compress their exercise into fewer days, often during weekends or on 1 day in the middle of the week [44]). During the A1 and A2, participants were asked not to use the ‘MyDayPlan’ app and to wear a Fitbit activity tracker with the screen covered. This because the Fitbit is also used as an outcome measure instrument in this study, next to an intervention component for self-monitoring. During B1 and B2, participants were asked to use the ‘MyDayPlan’ app and to wear a Fitbit activity tracker without a screen cover.

**Fig. 1:**
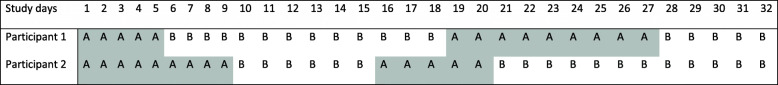
Specific ABAB reversal design (2 participants)

Prior to the start of the study (during the week before the study) all participants were visited at home. During this visit, participants were provided information (purpose of the study, content of the study, expectations of the participants, procedure, risks and advantages, termination of participation, incentives, confidentiality and contact information) about the study. This was done by going through an information letter together. Afterwards they were invited to provide written informed consent. During this home visit participants were also provided with a Fitbit, charging cable and stickers to cover the screen of the Fitbit. To minimize participant burden and to minimize technological difficulties at the start of the study, the Fitbit app was installed on their smartphone and the Fitbit was paired to it. Furthermore, the ‘MyDayPlan’ app was installed on their phone and phone settings were checked to allow notifications from the app. However, they did not yet log into the app. To avoid bias during the A1, no substantive information about the content of the app was given during this home visit. The study started 2 days after the home visit, giving the participants the opportunity to fully charge the Fitbit. A day before the start of the study, participants were provided with a login for the MyDayPlan app by e-mail. They were asked to log into the app on the following morning. The day of the first log-in was automatically labelled as the first day of the study. From that moment on, the ‘MyDayPlan app’ provided all communication with the participant. For the coming 32 days, the participant did not know in advance when to use or not use the ‘MyDayPlan’ app. Therefore, each morning, participants received a notification with the message to either use the app that day or not to use the app that day. When they were required not to use the app, they were also required to cover the screen of the Fitbit with the sticker they received during the home visit. After testing various strategies to avoid self-monitoring using the Fitbit but still wear the Fitbit as an outcome measure instrument during the A phases, covering the screen was evaluated as the most feasible and effective strategy. In Fig. 1 the design of two random participants is shown as an example.

After completing the 32 days of study, a second visit was scheduled to collect the Fitbit and the charging cable.

### Measurement

#### Participant characteristics

Demographic characteristics (age, gender, residence, and profession) and current level of physical activity of participants were assessed using an online questionnaire, which was sent to the participants via email. The questionnaire included the long version of the International Physical Activity Questionnaire (IPAQ) (translated into Dutch) [45]. The IPAQ assesses self-reported PA during the past week in 4 domains (work, transport, household, and leisure time) and provides indicators for the amount of work-related PA, transport-related PA, household-related PA, leisure-time PA, total PA, vigorous-intensity PA (VPA), and moderate-intensity PA (MPA) per week. The IPAQ has good reliability (intraclass range 0.46–0.96) and a fair-to-moderate criterion validity (Spearman rho between .30 and .37) [46]. As the IPAQ overestimates physical activity [47], the data were truncated according to the method described by Dubuy et al. [48].

#### Steps

The Fitbit Charge was used to assess participants’ daily number of steps as a primary outcome. Fitbit Charge (Fitbit Inc., San Francisco, CA) is a wireless, wrist-worn, triaxial accelerometer. A proprietary algorithm translates raw acceleration signals into steps and activity levels [49]. It estimates steps, activity level, and energy expenditure each minute. The Fitbit Charge has shown to be a reliable (intraclass range 0.69–0.87) and valid (Spearman Rho between .80 and .96) measure for measuring daily number of steps, compared to direct observation and ActiGraph GT3X+ measurements in adults [41, 50]. Participants were instructed to wear the Fitbit, in accordance with manufacturers’ guidelines, on the non-dominant wrist. The Fitbit device was worn during all waking hours, except during water-based activities. Only valid days were included, and these were defined as days with ≥10 h of wear time [51]. Non-wear time was identified as bouts of ≥60 consecutive minutes with zero steps [52].

### Statistical analysis

The data of the individual participants were analyzed with multilevel modeling to estimate the average intervention effect of ‘MyDayPlan’ across 35 cases (i.e. aggregated single case data, see Van den Noortgate & Onghena, 2007) [53]. All analyses were conducted using R version 3.2.5 [54]. To take into account the hierarchical nature of the data (measurement occasions nested within cases: several days of step data within the same participant) two-level models were fitted (for a similar approach, see [55]). To estimate the variability in step counts within versus between individuals in the baseline phase, a random intercept null model was fitted. To test the two main hypotheses, three different models were fitted. Model A was used to test the first hypothesis which states that the use of ‘MyDayPlan’ will increase the number of daily steps and that the number of daily steps will decrease when participants stop using the app. Model B and C were used to test the second hypothesis which states that a carry-over effect will be seen when participants stop using the app. More specifically, model B evaluates whether the number of daily steps differs significantly between A1 and A2 which could already be an indication of the presence of a carry-over effect. Model C evaluates whether the trend in the first and second control phase differ significantly as this could also indicate the presence of a carry-over effect. The trend in a particular phase refers specifically to the slope of the linear trendline that is drawn based on all data points within that phase. Furthermore, It is important to indicate that model A evaluates a possible *average* intervention effect whereas model C evaluates a possible *immediate* intervention effect. The immediate intervention effect refers to the immediate change (increase/decrease) in number of daily steps, the first day the intervention is presented. Whereas the average intervention effect refers to the average change (increase/decrease) in number of daily steps during the period of days the intervention is presented. The used models are described in more detail as supplementary material (supplementary file 2).

## Results

### Demographic characteristics

All 35 participants completed the study. Characteristics (sex, age and moderate-to-vigorous physical activity (MVPA) level) of these 35 participants are presented in Table 2. Furthermore, all participants achieved 32 days of valid wear time of the Fitbit.

**Table 2:** Participants’ characteristics

Characteristics	Participants (*N* = 35)
**Sex, n (%)**	
Men	14 (40)
Women	21 (60)
**Age in years, mean (SD), range**	40 (13,2), 18–58
**MVPA in min per day, mean (SD), range**	19,9 (8,7), 0–29,8

### Results of the multilevel models examining the effects of MyDayPlan on daily steps

**Fig. 2:**
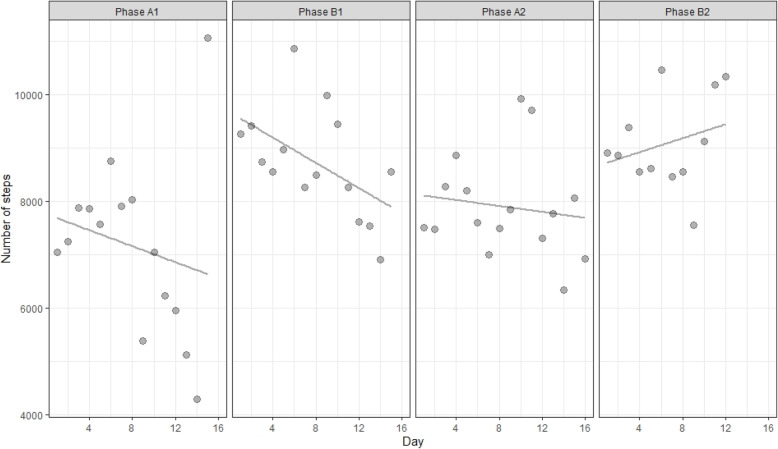
Graphical display of the ABAB reversal design showing the average number of steps per day across participants

The average number of steps per day across is depicted in Fig. 2. Furthermore, the average, median, minimum and maximum number of steps per phase is also presented in Table 3. Figure 2 also visualizes the slope within a phase. On the x-axis of Fig. 2, the various chronological days of the four phases are presented (from the first to the sixteenth day). Each dot on the figure represents the average number of steps for all participants on that particular day. Decomposing the variability in A1 in step counts between and within individuals demonstrated that the majority of the variability in step counts was observed within individuals (59%).

**Table 3:** Average (SD), minimum and maximum number of steps per phase

Phase	Average (SD)	Median (IQR)	Minimum	Maximum
Baseline phase (A1)	7461 (3901)	7308 (4742)	895	21,648
Intervention phase (B1)	9052 (3731)	8657 (4278)	1268	25,192
Reversal phase (A2)	7951 (4106)	7274 (5301)	807	26,081
Intervention phase (B2)	8986 (4142)	8754 (4340)	1346	33,778

#### Model a: change in outcome score when new phase is introduced

The average number of steps increased significantly from the control to intervention phases for each AB combination. From A1 to B1, the model revealed an average increase of 1424 steps (95% CI [775.42, 2072.32], t (1082) = 4.31,*p* < .001) From A2 to B2, the model revealed an average increase of 1181 steps (95% CI [392.98, 1968.16], t (1082) = 2.94, *p* = .003). The average change in level from B1 to A2 was also significant (95% CI [− 1755.60, − 512.38], t (1082) = − 3.58, *p* < .001), indicating that the number of steps decreased significantly (1134 steps) when going from the first intervention phase to the second control phase.

#### Model B: difference between baseline conditions (A1 and A2) and intervention conditions (B1 and B2)

The difference in the average number of steps between the first and the second control phase was not statistically significant. (During A2, participants took on average 289 steps more than during A1, 95% CI [− 374.71, 951.87], t (1082) = 0.85, *p* = .39). Likewise, the difference in the average intervention effect between the second and the first AB pair was not significantly different (decrease of 282 steps, 95% CI [− 1186.14, 622.41], t (1082) = −.61, *p* = .54).

#### Model C: is there a difference in slope between consecutive phases and what is the difference in slope between common phases?

In contrast to Model A in which the average intervention effect is examined, in Model C, the immediate intervention effect is examined. This effect refers to the immediate change in outcome score (number of daily steps) the first day after the ‘MyDayPlan’ intervention was presented. The immediate intervention effect during the first AB pair was statistically significant, revealing an increase of 1544 steps (95% CI [580.40, 2507.41], t (1078) = 3.14, *p* = .002). However, the immediate intervention effect during the second AB pair, an increase of 888 steps, was not significant (95% CI [− 210.05, 1985.93], t (1078) = 1.59, *p* = .11). There was a significant effect of removing the intervention, more specific a decrease of 1162 steps (95% CI [− 2115.70, − 208.99], t (1078) = − 2.39, *p* = .02), immediately after removing the intervention. We found no evidence for a trend in the first baseline (95% CI [− 137.76, 111.95], t (1078) = −.20, *p* = .84) and intervention phase (95% CI [− 137.41, 99.93], t (1078) = −.31, *p* = .76), nor did we find evidence for a difference in slope between the two control (95% CI [− 114.59, 197.99], t (1078) = .52, *p* = .60) and intervention phases (95% CI [− 128.79,284.22], t (1078) = .74, *p* = .46). Although statistically there may not be a difference between the two interventions phases, visually, it seems that B1 has a negative slope whereas B2 shows a positive slope. The fact that the amount of daily steps increases towards the end of B2, could possibly be explained by the fact that participants knew or assumed that the end of the study was near. As a reaction to this, participants could have engaged in more physical activity.

## Discussion

### Effectiveness of MyDayPlan

This study investigated the effect of a self-regulation-based mHealth intervention, MyDayPlan, on the number of daily steps in a general adult sample. Hypotheses were tested using a single-case ABAB reversal design with 35 participants [56]. Analyses were done using a multilevel (two-level) model allowing to aggregate single case-results and estimate the intervention effect across all participants [55].

The analyses revealed that the use of ‘MyDayPlan’ has a positive and significant effect on the number of daily steps. More specifically, the number of daily steps increased during the periods that participants used the ‘MyDayPlan’ app, but also decreased when support of the ‘MyDayPlan’ app was no longer available. An average increase of respectively approximately 1500 and 1000 steps per day was measured during both intervention phases. This corresponds to an increase of respectively 19 and 15%. Earlier research that directly measured the number of steps and verified activity intensity in absolute terms of metabolic equivalents or METs (1 MET = 3.5 ml O2/kg/min or 1 kcal/kg/hour) have concluded that, despite individual variation, a cadence of 100 steps/minute represents a reasonable heuristic value for moderate intensity physical activity [57, 58]. If the extra steps in this study were taken at a cadence of 100/min this would mean an increase of 14 and 12 min of moderate-intensity PA per day respectively, resulting in an increase of 80 to 100 min MVPA per week. This is a substantial part of the 150 min of MVPA per week stated by the health guidelines. Other research, of Aoyagi and Shepard revealed that each 1000 step increment in daily free-living activity up to 6000 steps/day was associated with an additional 2.5 min of MVPA. From 6000 to 12,000 steps/day each 1000 step increment added another 5 min of MVPA [59]. Furthermore the found effects are congruent with the accepted concept that some activity is better than none, and that some relatively important health benefits may be realized with improvements over the lowest levels [60]. This positive intervention effect on PA is in line with earlier self-regulation based interventions using e- and mHealth revealing increases in MVPA of 16 to 67% depending on the target population and whether self-reported or accelerometer-based assessment of MVPA was used [26–29]. The findings of this study are also in line with the findings of a review of Cugelman et al., revealing that online interventions which help users to set and achieve goals, teach them skills, and offer services to track and report users’ progress toward their goals show positive effects on physical activity levels [61]. Of note, these interventions used self-regulation cycles that were long (weekly). This study also revealed that more than half of the variability in number of daily steps in normal circumstances (during A1, the first control phase) is mainly not due to differences between participants, but variability within a person. This finding emphasizes the importance of day-to-day interventions. Factors fluctuating on a daily level probably influence daily physical activity, so it is important to adjust goals, action and coping plans accordingly.

Furthermore, the used ABAB reversal design allowed to also look at the evolution of the behaviour over time and the effect of withdrawing the intervention on the level of physical activity, more specifically the number of daily steps. To the authors knowledge this is the first study looking at a carry-over effect after fully withdrawing the intervention. In contrast with the hypothesis, no carry-over effect was revealed. In the current study, it was hypothesized that, after a period of using the app, the number of daily steps would still be higher during the first few days after removing the ‘MyDayPlan’ app compared to baseline level. This hypothesis was based on the idea that, after ‘mandatory’ exposure to the self-regulation techniques of ‘MyDayPlan’, users potentially would adopt techniques such as action and coping planning even without using the app. However, this was not found in this study. This is in contrast with two earlier studies using single case designs to test effectiveness of self-regulation techniques to increase walking. However, these studies did not adopt an ABAB reversal design with a full withdrawal of the intervention. They instead revealed a cumulative carry-over effect, meaning that the effect on target behaviour (increase in physical activity) increases over time. These results suggest the potential of self-regulation techniques for carry-over effects across conditions [62, 63]. The fact that no carry-over effect was revealed in the current study might be explained by the fact that the ‘exposure period’ was too short to adopt self-regulation techniques such as action and coping planning as a new daily routine without guidance. A second explanation could be that the transition between a period of using ‘MyDayPlan’ in which guidance is provided while setting goals, action and coping plans and a period of non-usage of the app, in which participants are left to their own devices, is too large. A possible solution could be the implementation of a period in which the app is not fully used, but for example each morning and each evening only a prompt is provided to remind users to engage in goal setting, action planning, coping planning, self-monitoring and goal reviewing. Therefore, future research should further investigate this possible carry-over effect by searching for the optimal ‘exposure period’ and the potential of gradually phasing out the self-regulation guidance to maximize the carry-over effect on the short and long term. Ideally, a possible carry-over effect could indicate that the self-regulation cycle has become more of a habit making the use of the app superfluous. Furthermore, for ‘MyDayPlan to be really valuable for public health, it should be able to cause long term effects on physical activity levels. However, aiming for long term effect of the app should not automatically mean lifelong using it. When temporary using the app, ‘MyDayPlan’ could act as a framework providing users with essential cognitive skills (goal setting, action planning, coping planning) leading to an increase in physical activity. After this ‘exposure period’, users could have mastered those skills, being able to execute them without guidance of ‘MyDayPlan’. This could be the key to ensure long term effects of the ‘MyDayPlan’ intervention. Contributing to this could be the implementation of a ‘transition period’ in which the app is not fully used, but for example each morning and each evening only a prompt is provided to remind users to engage in goal setting, action planning, coping planning, self-monitoring and goal reviewing.

### Strengths and limitations

This study has several strengths. First, this is one of the first mHealth interventions implementing a daily self-regulation cycle, allowing the users to set short term action and coping plans. This leads to a more dynamic intervention, making it possible to anticipate fluctuating contextual and psychosocial factors. Second, this is one of first studies using an ABAB reversal design to test effectiveness of a physical activity intervention, and more specifically of an mHealth intervention. This design allows to individually vary the length of the phases to avoid influence of the start day of the different phases and of the weekly physical activity pattern that is typically shown (e.g. the so called ‘weekend warriors’ who compress their exercise into fewer days, often during weekends or on 1 day in the middle of the week [44]). As described in the methods section, this design has several advantages over a more classic experimental design such as a randomized controlled trial design: it lends itself more to closely investigate a possible intervention effect on two moments and a possible carry-over effect after withdrawal of the intervention during the reversal phase. In combination with the used multilevel analysis, estimations can be made and conclusions can be drawn on the level of the group across all participants. Furthermore, fewer participants are needed with this design to achieve the same statistical power compared to an RCT design, because of the large number of repeated measurements within participants. A third strength of this study is the use of the Fitbit Charge as an outcome measure instrument instead of using an extra measuring instrument, such as the ActiGraph accelerometer. The double function of the Fitbit, being part of the ‘MyDayPlan’ intervention as a self-monitoring tool and also being used as an outcome measure instrument minimizes participant burden, increasing adherence to the intervention. Fourth, this study only included participants who did not meet the weekly physical activity recommendations of 150 min of weekly MVPA, and therefore belong to the target population, which benefits from physical activity interventions. Fifth, the evaluation of possible carry-over effect after the withdrawal of the intervention, contributes to exploring a possible long term effect of the intervention. Finally, although e- and mHealth interventions are often characterized by large attrition, no attrition was shown during this study of 32 days, indicating high user engagement with ‘MyDayPlan’. However, future research should further look into user engagement with ‘MyDayPlan’. Furthermore, session completion rates were not 100%, but were still quite high. Some participants used the same action and coping plan for consecutive days and some participants only used the proposed action and coping plans, making it very easy and little time-consuming to complete all the sessions. It could be assumed that this has contributed to the high completion rates.

There are also a number of limitations. First, the goal of this study was to maximize the intervention effect by providing the full ‘MyDayPlan’ intervention, consisting of a bundling of various self-regulation techniques during the intervention phases, and providing nothing during the control phases. Although this study revealed that the use of ‘MyDayPlan’ has a significant positive effect on the level of physical activity, this effect can only be attributed to the entirety of the ‘MyDayPlan’ intervention. Within this study it was not explored whether these techniques weigh contributed equally to the revealed effect, or whether a limited set/particular combination of these techniques is as effective or even more effective. Gaining insight into the efficacy of the various self-regulation techniques within the intervention could therefore be important for future research with ‘MyDayPlan’ [64]. Furthermore, in the current version of ‘MyDayPlan’, information for example on how many participants achieved their daily step goal and on how many days and on whether or not they inserted much variation in their action and coping plans, was not available. This detailed quantitative and qualitative information on how participants give substance to goal setting, action and coping planning is missing. Future research should definitely take into account this information to further understand and optimize the possible contribution of ‘MyDayPlan’ in increased physical activity levels. Another limitation is that this study did not evaluate the effect of ‘MyDayPlan’ on the potential mediators and moderators (e.g., factors such as attitudes, self-efficacy, and skills) that might explain the increase in number of daily steps [65, 66]. Behaviour change is assumed to be the result of changes via behavioural or environmental determinants [67], and the implemented self-regulation techniques of ‘MyDayPlan’ are expected to have an impact on such determinants, so it is important to also understand the change mechanisms [68]. Future research should investigate the effect of ‘MyDayPlan’ on these potential mediators and moderators of physical activity. Also, using number of daily steps as an outcome measure makes it difficult to translate the revealed effects of this study into progress towards the current MVPA health guidelines of WHO and the potential health benefits associated with these [3]. A final limitation of this study is the lack of power analysis to calculate the required sample size for this study. Sample size was mainly determined on the basis of previous, similar research. Performing the required sample size calculations could have strengthened the revealed findings. However, we assumed that the power, despite not being statistically determined, was sufficient to support the conclusions made. In addition, no post-hoc power analyses were performed because there were no indications that the effects found were under-powered. The effects found could always be adequately interpreted and explained.

## Conclusions

This study adds evidence that the self-regulation mHealth intervention, ‘MyDayPlan’ has the capacity to positively influence physical activity levels in the short term in a general adult population. More generally, this provides some first evidence for the potential of interventions adopting a daily self-regulation cycle, allowing for more short term action and coping plans. Given the high reach and low cost of online technologies, this could potentially help individuals towards adopting a physically active lifestyle. However, no carry-over effect was revealed when the app was no longer used. This could be attributed to the short ‘exposure period’ or the sudden transition between full use and no use. As a carry-over effect might be essential to ensure long term effects on the level of physical activity, this should be subject to future research with ‘MyDayPlan’.

## Supplementary Information

**Additional file 1.**

**Additional file 2.**

**Additional file 3.**

## Data Availability

The datasets used and/or analysed during the current study are available from the corresponding author on reasonable request.
