# Supplementary material for: Effectiveness of the mHealth intervention ‘MyDayPlan’ to increase physical activity: an aggregated single case approach
Source: Int J Behav Nutr Phys Act. 2021 Jul 7;18:92. doi: 10.1186/s12966-021-01163-2 (PMC8265041; doi:10.1186/s12966-021-01163-2)
Supplement: Supplementary file 1 — Additional file 1. [file 12966_2021_1163_MOESM1_ESM.pdf]

Supplementary File 1: screenshots of ‘MyDayPlan’

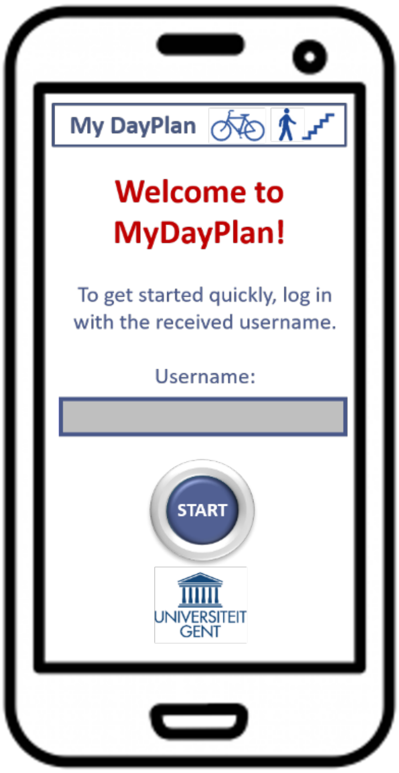

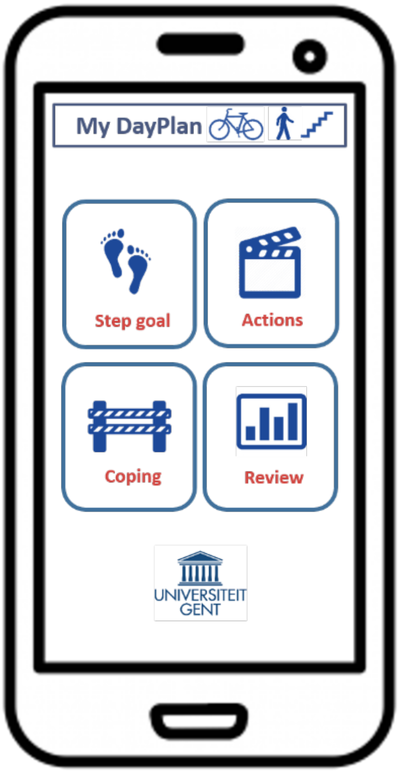

Goal Setting (Morning)

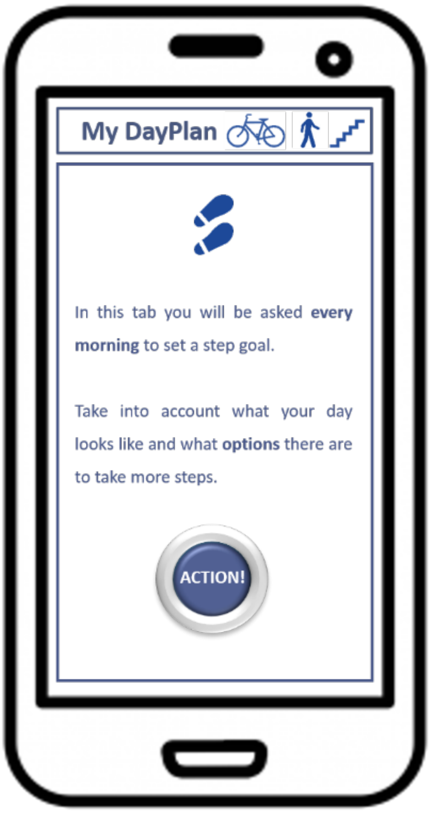

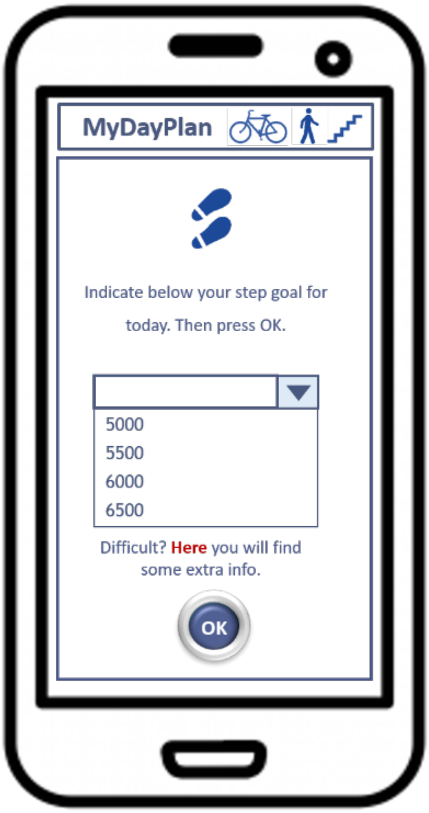

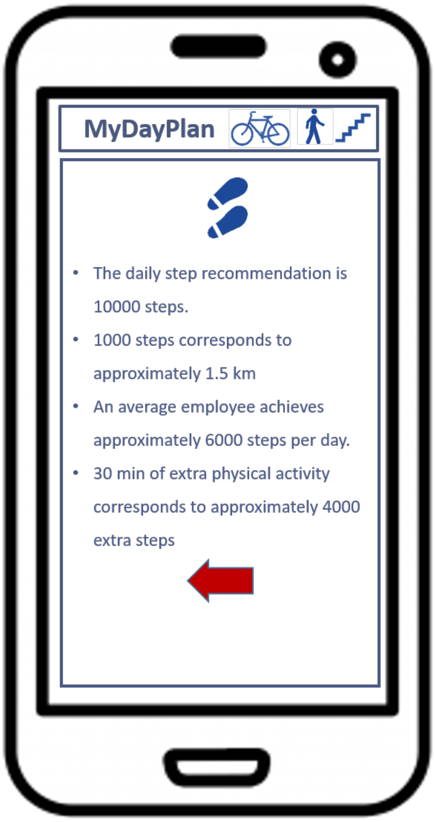

Action Planning (Morning)

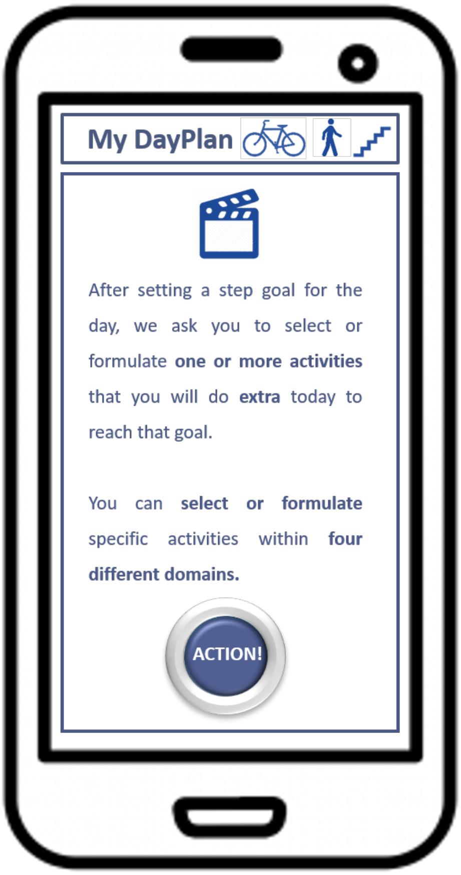

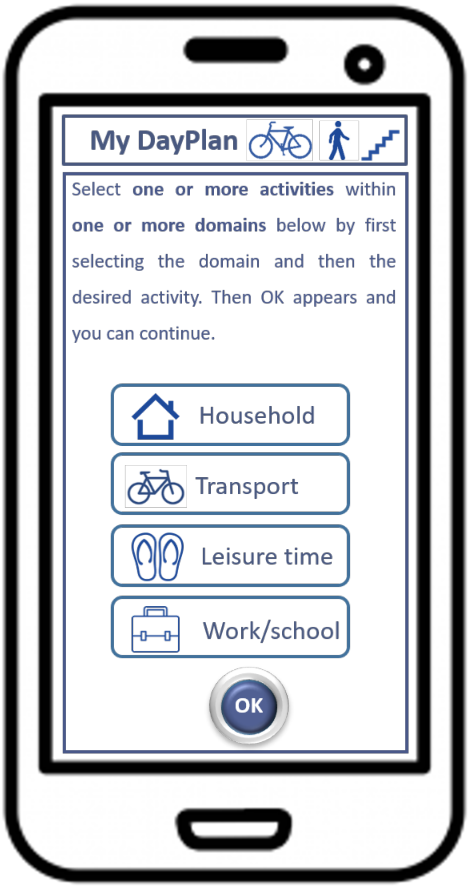

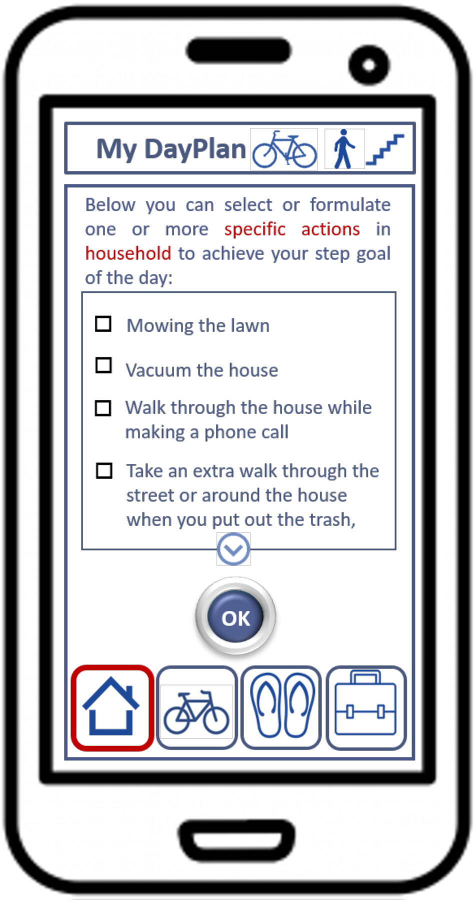

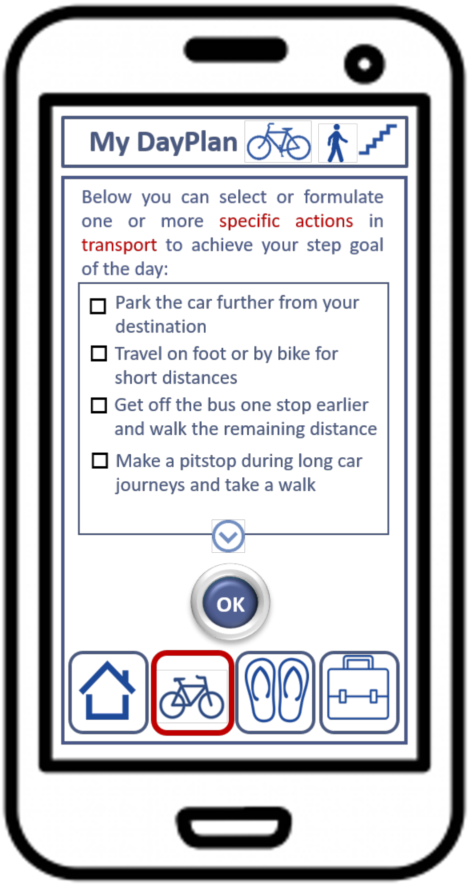

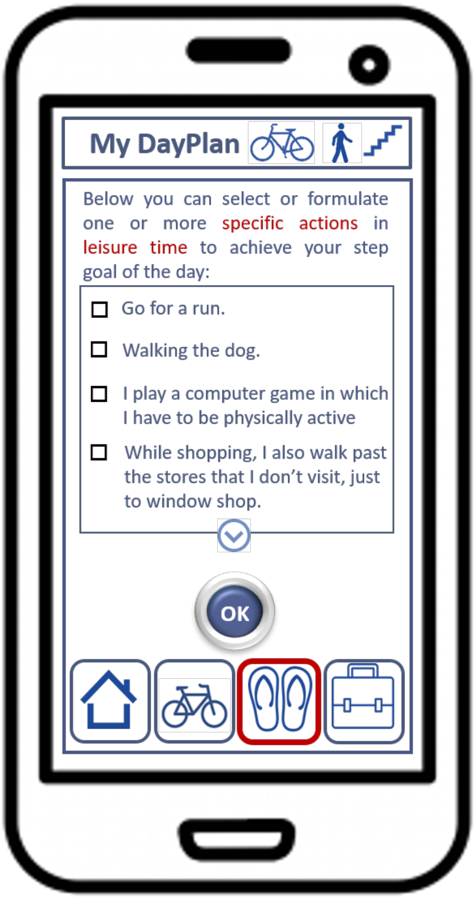

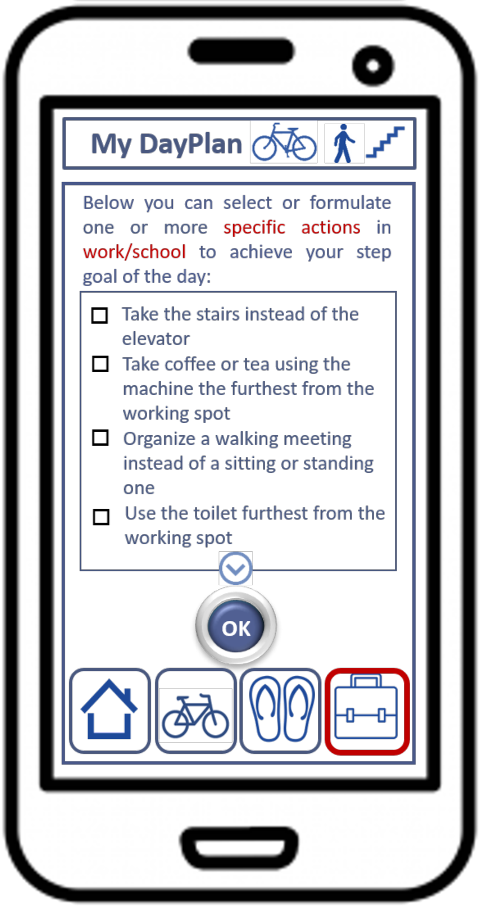

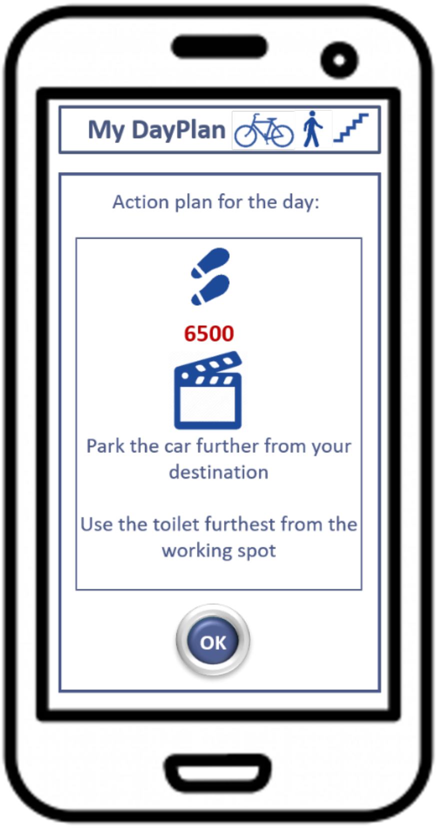

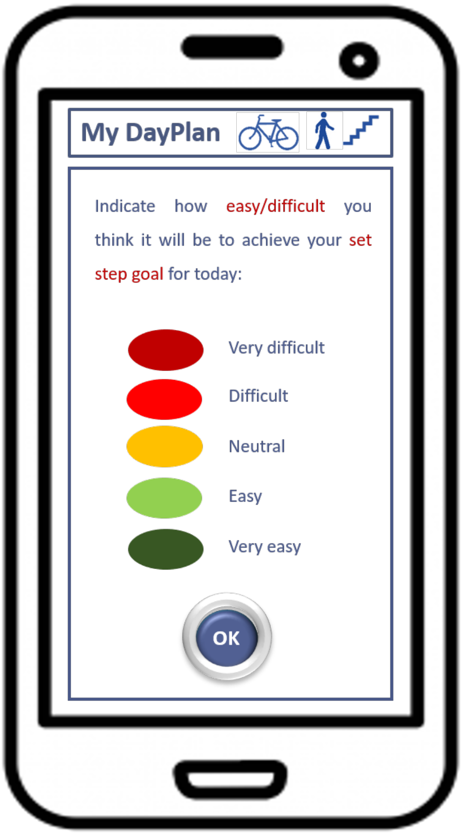

Coping Planning (Morning)

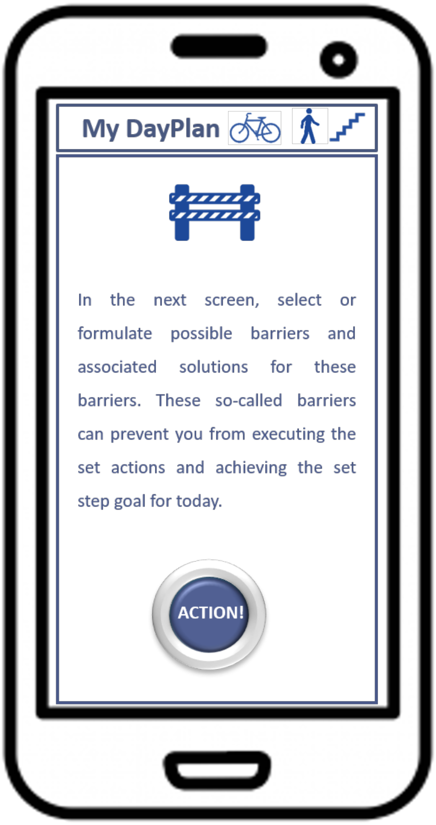

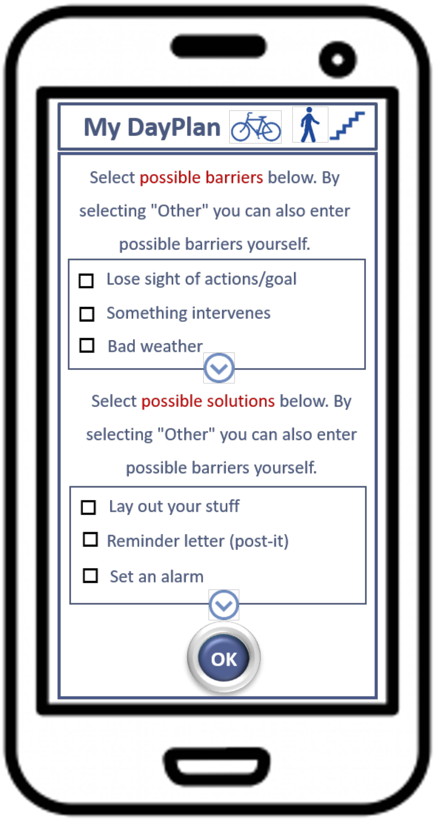

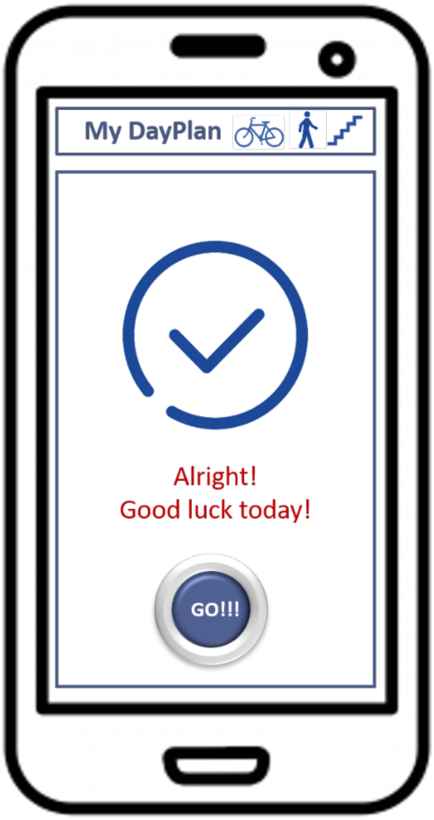

Prompting review of behavioural goals

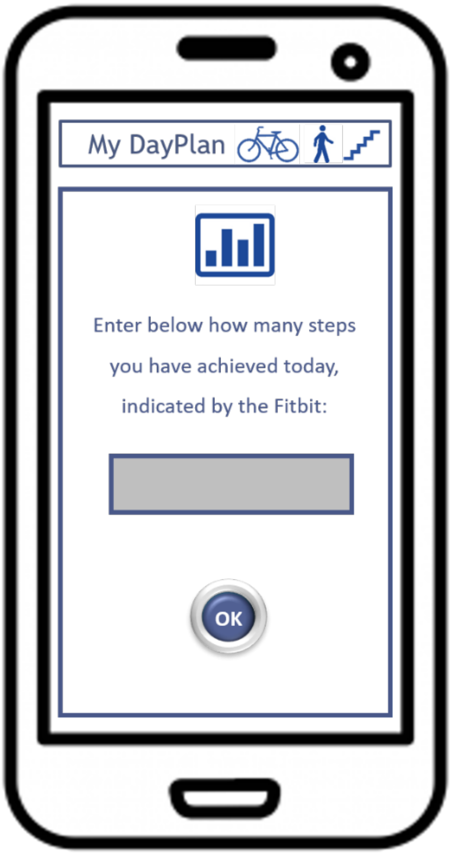

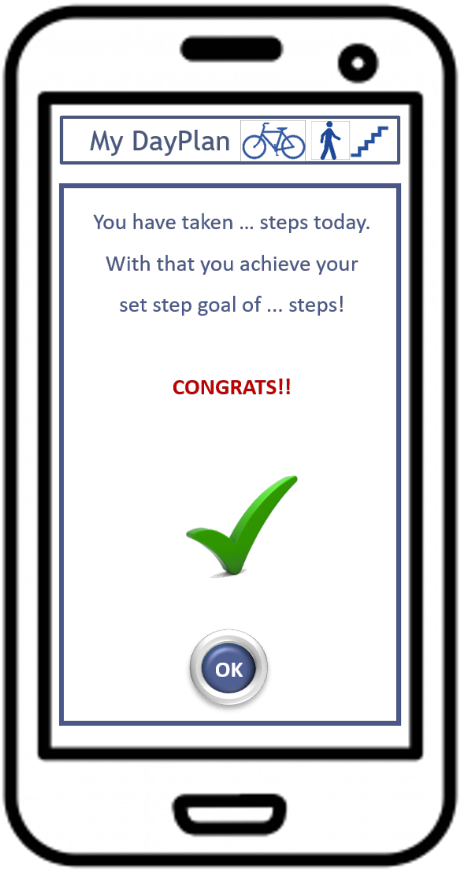

OR

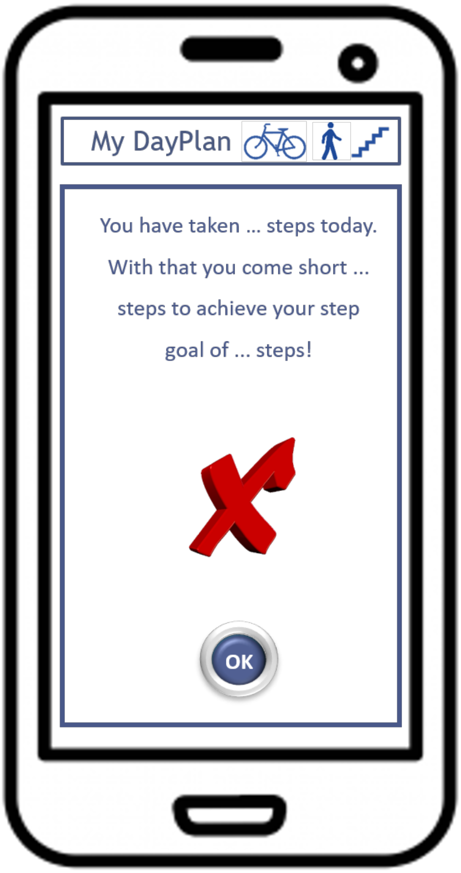

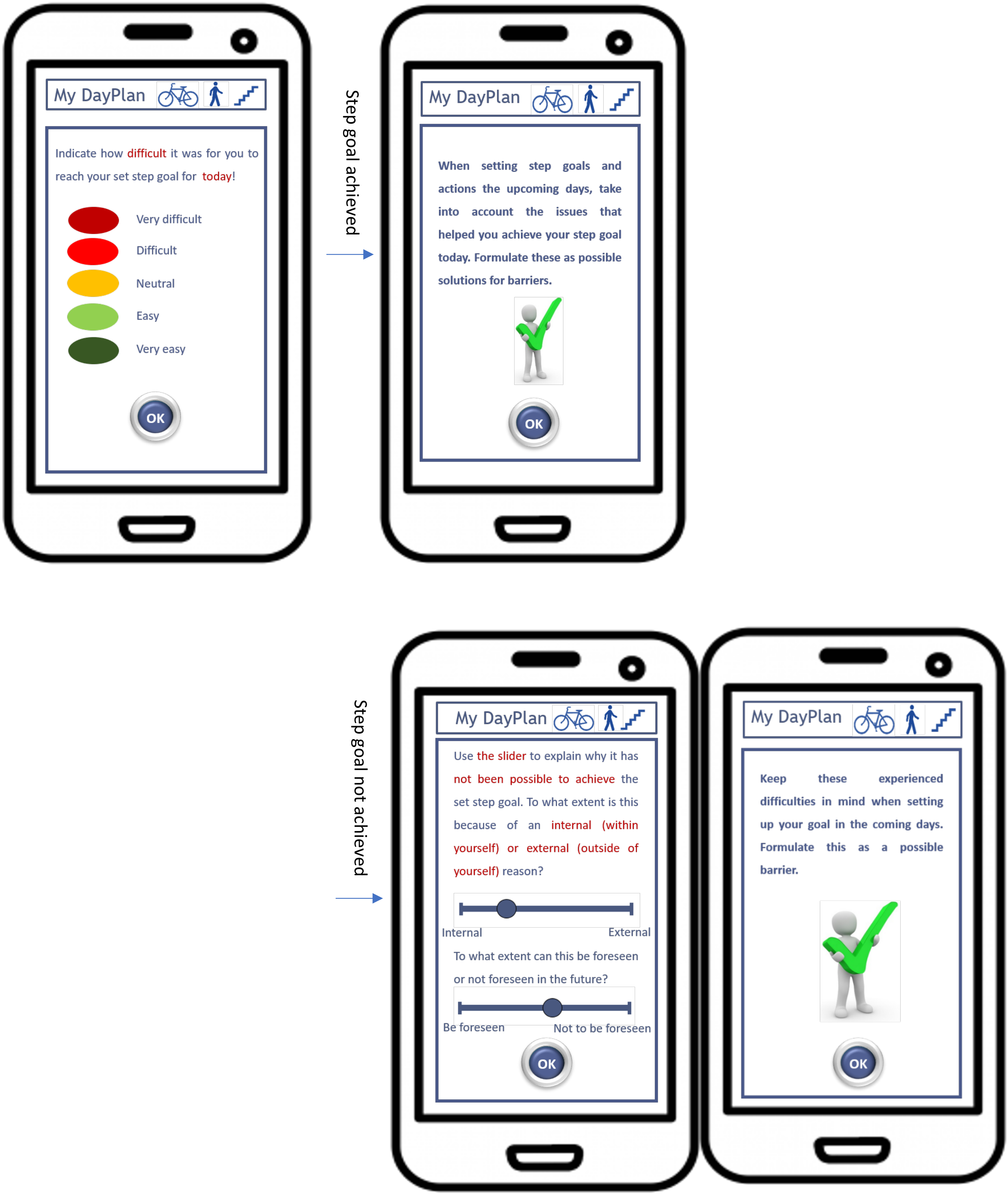
My DayPlan
Indicate how difficult it was for you to reach your set step goal for today!
Very difficult
Difficult
Neutral
Easy
Very easy
OK
Step goal achieved
My DayPlan
When setting step goals and actions the upcoming days, take into account the issues that helped you achieve your step goal today. Formulate these as possible solutions for barriers.
OK
Step goal not achieved
My DayPlan
Use the slider to explain why it has not been possible to achieve the set step goal. To what extent is this because of an internal (within yourself) or external (outside of yourself) reason?
Internal
External
To what extent can this be foreseen or not foreseen in the future?
Be foreseen
Not to be foreseen
OK
My DayPlan
Keep these experienced difficulties in mind when setting up your goal in the coming days. Formulate this as a possible barrier.
OK

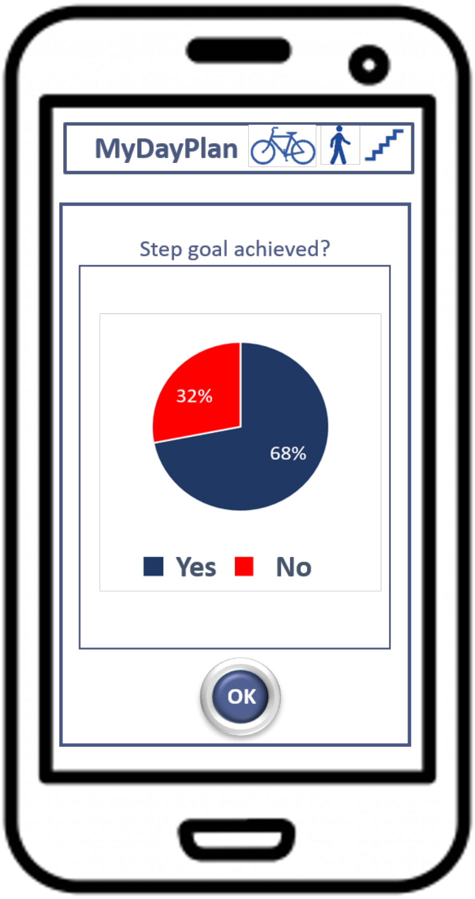
MyDayPlan
Step goal achieved?
32%
68%
Yes
No
OK
