# Supplementary material for: Effectiveness of the mHealth intervention ‘MyDayPlan’ to increase physical activity: an aggregated single case approach
Source: Int J Behav Nutr Phys Act. 2021 Jul 7;18:92. doi: 10.1186/s12966-021-01163-2 (PMC8265041; doi:10.1186/s12966-021-01163-2)
Supplement: Supplementary file 2 — Additional file 2. [file 12966_2021_1163_MOESM2_ESM.pdf]

## Supplementary File 2: Multilevel modelling (Model A,B and C)

It is likely that outcome scores that are measured closer in time (number of steps measured at days closer to each other) are more related to each other than outcome scores measured further away in time. Moreover, there might be more variability in the outcome (=number of daily steps) scores during the intervention phases in comparison to the baseline phases, or vice versa. Therefore, we compared all models described above with a model with a first-order autoregressive covariance structure for the within-participant variance and a model with heterogeneous phase variances. Models were compared with likelihood ratio tests.

*Model A*

To estimate the change in the number of daily steps (=outcome score) when another phase (A1/B1/A2/B2) was introduced, the following model was fitted:

Level 1 $$y_{ij} = \beta_{0j} + \beta_{1j}A1B1_{ij} + \beta_{2j}B1A2_{ij} + \beta_{3j}A2B2_{ij} + e_{ij} \qquad e_{ij} \sim N(0, \sigma_e^2) \quad (1)$$

Level 2
$$\beta_{0j} = \theta_{00} + u_{0j}$$
$$\beta_{1j} = \theta_{10} + u_{1j}$$
$$\beta_{2j} = \theta_{20} + u_{2j}$$
$$\beta_{3j} = \theta_{30} + u_{3j}$$

$$\begin{bmatrix} u_{0j} \\ u_{1j} \\ u_{2j} \\ u_{3j} \end{bmatrix} \sim \mathrm{N}\left( \begin{bmatrix} 0 \\ 0 \\ 0 \\ 0 \end{bmatrix}, \begin{bmatrix} \sigma_{u_0}^2 & \sigma_{u_0u_1}^2 & \sigma_{u_0u_2}^2 & \sigma_{u_0u_3}^2 \\ \sigma_{u_1u_0}^2 & \sigma_{u_1}^2 & \sigma_{u_1u_2}^2 & \sigma_{u_1u_3}^2 \\ \sigma_{u_2u_0}^2 & \sigma_{u_2u_1}^2 & \sigma_{u_2}^2 & \sigma_{u_2u_3}^2 \\ \sigma_{u_3u_0}^2 & \sigma_{u_3u_1}^2 & \sigma_{u_3u_2}^2 & \sigma_{u_3}^2 \end{bmatrix} \right) \quad (2)$$

In equation (1) $y_{ij}$ is the number of steps a participant *j* has taken on day *i*. Dummy variables $A1B1_{ij}, B1A2_{ij}$ and $A2B2_{ij}$ are used to indicate the phase (1). $A1B1_{ij}$ equals 1 if measurement occasion *i* from participant *j* is obtained after the first baseline phase; $B1A2_{ij}$ equals 1 for all measurement occasions after the first intervention phase and $A2B2_{ij}$ equals 1 if the measurement occasion occurs in the last intervention phase. If $A1B1_{ij}$, $B1A2_{ij}$ and $A2B1_{ij}$ equal 0 simultaneously, then the measurement is taken in the first baseline phase (A1).

In equation (2) $\theta_{00}$ indicates the average baseline level and $\theta_{10}, \theta_{20}$ and $\theta_{30}$ represent the change in outcome score when phase B1, A2 and B2 respectively are introduced across the J participants. $u_{0j}$, $u_{1j}, u_{2j}$ and $u_{3j}$ are the participant-specific residuals indicating how much each individual participant deviates from the average effects. Both the level 1 and level 2 residuals are assumed to be independent, identically and (multivariate) normally distributed.

The model taking into account autocorrelation was significantly better than the model that assumed independent within-participant errors (-2LL = 25.23, $p$ < .001). Allowing heterogeneous phase variance did not further improve the fit of the model (-2LL = 3.53, $p$ = .62). The final model has an autoregressive covariance structure with homogenous within-participant variances, meaning that the variance within the intervention phases did not differ significantly from the variance within the control phases. Parameter and standard error estimates for the final model are presented in Table 1.

Table 1. Parameter and standard error estimates from estimation of Model A (1).

| | Parameter | Parameter estimate | *SE* | *p* |
|---|---|---|---|---|
| Average baseline level, first AB pair | $\theta_{00}$ | 7600.98 | 486.56 | <.001 |
| Average intervention effect, first AB pair | $\theta_{10}$ | 1423.87 | 330.48 | <.001 |
| Average change in level, from B1 to A2 | $\theta_{20}$ | -1133.99 | 316.80 | <.001 |
| Average intervention effect, second AB pair | $\theta_{30}$ | 1180.57 | 401.39 | .003 |

*Model B*

To compare the score for the two baseline conditions (A1 and A2) and the two intervention conditions (B1 and B2), a two-level model was fitted with *pair* (first AB pair vs. second AB pair) and *phase* (A phase versus B phase) as predictors (model B). In equation (3) the dummy variable , $Pair_{ij}$, indicates whether the measurements belong to the first or the second AB pair. $Pair_{ij}$, equals 1 if the measurement occasion belongs to the second AB pair, zero otherwise. The dummy variable $Phase_{ij}$ indicates whether measurement *i* is part of the baseline phase (A1/A2) or the intervention phase (B1/B2). If the measurement occasion belongs to B1 or B2, then $Phase_{ij}$ equals 1, otherwise zero (2).

Level 1 $$y_{ij} = \beta_{0j} + \beta_{1j}Pair_{ij} + \beta_{2j}Phase_{ij} + \beta_{3j}Pair_{ij}Phase_{ij} + e_{ij} \qquad e_{ij}\sim N(0,\sigma_e^2) \quad (3)$$

The model taking into account autocorrelation was significantly better than the model that assumed independent within-participant errors (-2LL = 32.57, $p$ < .001). Allowing heterogeneous phase variance (model C) did not further improve the fit of the model (-2LL = 1.44, $p$ = .23). The final model has an autoregressive covariance structure with homogenous within-participant variances. Parameter and standard error estimates for this model are presented in Table 2.

Table 2. Parameter and standard error estimates from estimation of Model B (3).

| | Parameter | Parameter estimate | SE | p |
|---|---|---|---|---|
| Average baseline level, A1 | $\theta_{00}$ | 7593.94 | 482.07 | <.001 |
| Difference in outcome score between A2 and A1 | $\theta_{10}$ | 288.58 | 338.04 | .39 |
| Average intervention effect, first AB pair | $\theta_{20}$ | 1435.74 | 348.90 | <.001 |
| Difference between the average treatment effect during the second AB pair and the first AB pair | $\theta_{30}$ | -281.86 | 460.86 | .54 |

*Model C*

It is possible that carry-over effects might be present in the second baseline phase. This means that, even without using the app, participants take more steps during A2. If this would be the case, we would expect a difference in slope in the first versus the second baseline phase. To compare the change in slopes between the two common phases (Difference in slope between A1 and A2 and between B1 and B2), model A was extended with four time variables (T1 to T4)(2). T1 equals 0 at the start of phase A1. During phase B1, T1 remains constant. During the A2 phase, T1 begins to increase again until the start of the B2 phase. This is because we want to compare the trend during the A2 phase with the trend during A1. A similar coding scheme is used for T2. T2 is centered around the start of B1, remains constant during phase A2 and continues to increase during the B2 phase. This is because we want to compare the trend during B2 with the trend during B1. T3 is centered around the start of the second baseline and is set constant during the second intervention. T4 is centered around the start of phase B2.

Level 1 $$y_{ij} = \beta_{0j} + (\beta_{1j}T1_{ij}) + (\beta_{2j} + \beta_{3j}T2_{ij})A1B1_{ij} + (\beta_{4j} + \beta_{5j}T3_{ij})B1A2_{ij} + (\beta_{6j} + \beta_{7j}T4_{ij})A2B2_{ij} + e_{ij} \qquad e_{ij} \sim N(0, \sigma_e^2) \quad (4)$$

The model taking into account autocorrelation was significantly better than the model that assumed independent within-participant errors (-2LL = 25.95, $p$ < .001). Allowing heterogeneous phase variance (model C) did not further improve the fit of the model (-2LL = 0.65, $p$ = .42). The final model has an autoregressive covariance structure with homogenous within-participant variances. Parameter and standard error estimates for this model are presented in Table 3.

Table 3. Parameter and standard error estimates from estimation of Model C (4).

| | Parameter | Parameter estimate | SE | p |
|---|---|---|---|---|
| Outcome at the start of A1 | $\theta_{00}$ | 7639.13 | 524.00 | <.001 |
| Linear trend during A1 | $\theta_{10}$ | -12.91 | 63.63 | .84 |
| Immediate intervention effect in the first AB pair | $\theta_{20}$ | 1543.91 | 491.04 | .002 |
| Linear trend during B1 | $\theta_{30}$ | -18.74 | 60.48 | .76 |
| Immediate difference in outcome score when removing the intervention | $\theta_{40}$ | -1162.34 | 485.87 | .02 |
| Difference in trend between A2 and A1 | $\theta_{50}$ | 41.70 | 79.65 | .60 |
| Immediate intervention effect in the second AB pair | $\theta_{60}$ | 887.94 | 559.58 | .11 |
| Difference in trend between B2 and B1 | $\theta_{70}$ | 77.71 | 105.24 | .46 |

1. Shadish WR, Kyse EN, Rindskopf DM. Analyzing data from single-case designs using multilevel models: new applications and some agenda items for future research. Psychol Methods. 2013;18(3):385-405.
2. Moeyaert M, Ugille M, Ferron JM, Beretvas SN, Van den Noortgate W. The influence of the design matrix on treatment effect estimates in the quantitative analyses of single-subject experimental design research. Behavior modification. 2014;38(5):665-704.
